# Life History Traits and Developmental Duration of the Yellow Coster *Telchinia issoria* Hübner, 1819 (Lepidoptera: Nymphalidae) Under Laboratory Conditions

**DOI:** 10.3390/insects17020216

**Published:** 2026-02-19

**Authors:** Liuliu Dong, Xin Yang, Xiaoxiao Jin, Xujie Liu, Min Gao, Jie Fang

**Affiliations:** School of Life Sciences and Medical Engineering, Anhui University, No. 111 Jiulong Road, Hefei 230601, China; 18256523078@163.com (L.D.); lexbur2580@163.com (X.Y.); 13966945337@163.com (X.J.); 19851630996@163.com (X.L.); 19855677866@163.com (M.G.)

**Keywords:** insect life history, *Telchinia issoria*, development, lepidoptera, species interactions

## Abstract

Understanding a species’ life history is essential for assessing its adaptability and resource trade-offs under environmental stress. Given their diversity and ecological significance, Lepidoptera represent an ideal model system for studying such adaptive responses. Under controlled laboratory conditions, we quantified the life history traits of *Telchinia issoria* and examined their associations with key abiotic factors—temperature, humidity, and light intensity—across all developmental stages. The results showed that: (1) the complete developmental duration from egg to adult was first quantified, establishing a crucial baseline for understanding its life history strategy; (2) the egg stage exhibited the highest survival rate, whereas the eighth-instar larval stage showed the lowest; and (3) correlations with abiotic factors differed markedly across stages, indicating stage-specific environmental sensitivity. Faster larval development may be associated with higher temperature, humidity, and light intensity; pupal development with high humidity and low light; and adult lifespan with low temperature, high humidity, and dim light. These findings advance our understanding of insect developmental plasticity, supporting more accurate population models and informing insect management and biodiversity conservation under climate change.

## Simple Summary

*Telchinia issoria* is distributed exclusively in continental Southeast Asia. Its distinctive morphology makes it a frequent subject in studies of biological mimicry. To systematically characterize its life history, we documented its complete development from egg to adult under controlled laboratory conditions, quantifying the total developmental duration and stage-specific survival rates. The results of this study reveal potential stage-specific association patterns: larval duration showed strong negative correlations with both mean temperature and humidity; pupal duration exhibited a moderate positive correlation with mean humidity; and adult lifespan was strongly negatively correlated with mean light intensity. These results delineate the stage-specific environmental dependencies of *T. issoria* and provide a basis for predicting how environmental change may affect insect population dynamics.

## 1. Introduction

Life history describes how an organism allocates reproductive investment across different ages or developmental stages. It integrates genetic, phenotypic, and environmental factors that influence survival and reproduction, thereby reflecting adaptive strategies to its habitat [1]. Current research on insect life history parameters often focuses on key individual traits—such as developmental duration and survival rate—as well as integrated life table parameters (e.g., net reproductive rate), which quantify changes in fitness under environmental stress. For example, a recent study on the citrus butterfly (*Papilio demoleus* Linnaeus, 1758) showed that its complete life cycle under laboratory conditions lasts about 41 days, with an adult stage averaging 4.5 days, illustrating its developmental duration and lifespan in a controlled setting [2]. Similarly, a field-based controlled experiment on monarch butterflies (*Danaus plexippus* Linnaeus, 1758) revealed that larval survival rate is highly sensitive to phenological synchrony, with values ranging from 0.8% to 9.5%, significantly affecting the population’s recruitment potential [3]. Studies on life histories can reveal trade-offs among development, reproduction, and survival under environmental pressures [4]. For example, studies on weight changes during eclosion in Lepidopteran insects show that weight loss is closely linked to their capital breeding strategy, egg maturation, and sexual dimorphism. This offers insights into the evolutionary mechanisms behind adult body size formation, reproductive allocation, and flight adaptation in this group [5]. Further research reveals that among boreal Lepidopterans in Northern Europe, combining phenological advancement with a northward range shift is a key adaptation to climate warming. Notably, only about 15% of species that employ both strategies show the strongest population growth trends. These findings provide a basis for assessing their climate adaptation potential and informing conservation strategies [6]. From an evolutionary perspective, life history research uses concepts like developmental programming and resource allocation to help predict how species respond to extreme environments and climate change. For instance, the Antarctic midge (*Belgica antarctica* Jacobs, 1900) adapts to polar conditions through a two-year life cycle that includes two overwintering diapause periods and synchronized summer emergence. Its early-instar larvae can also endure extreme stressors through dormancy [7]. In summary, systematically studying key life history traits—such as phenological responses, diapause characteristics, weight changes, body size, and reproductive allocation—can reveal how organisms adapt to environmental fluctuations. This provides a theoretical foundation and practical guidance for understanding mechanisms, predicting adaptations, and supporting management efforts.

As ectotherms, the key life history traits of insects—including developmental rate, survival, and fecundity—are strongly influenced by environmental conditions [8]. Among the abiotic factors, temperature plays a central role by directly regulating insect development through effects on metabolic rate, food utilization efficiency, and immune responses [9]. Classic models, including the Sharpe–Schoolfield equation, frame this relationship: development accelerates with warmth up to an optimum, then declines under thermally stressful extremes [10]. However, the impact of temperature on insect development is both stage-specific and species-dependent [11]. For instance, although high temperatures can accelerate larval development in *D. plexippus*, this benefit diminishes under extreme heat [12]. Moreover, developmental rate can be decoupled from fitness-related traits under thermal variation. This is evidenced by findings in monarch butterflies, where warming accelerated larval development but left survival and pupal mass unchanged [13], demonstrating a dissociation between thermal effects on development and overall fitness. Research shows that temperature affects population dynamics by influencing parameters such as survival, development time, and morphology [14]. These effects often act simultaneously across multiple developmental stages [15] and vary among species, populations, and developmental stages [16]. Consequently, it is crucial to move beyond single-factor temperature models and quantify the integrated effects of multiple abiotic variables—such as temperature, humidity, and light—across life stages, in order to realistically understand insect responses to environmental change.

Humidity regulates insect development through complex mechanisms, influencing traits such as pupal color polymorphism and early survival rates. Low humidity can induce the brown pupal morph (which may provide adaptive winter camouflage) and generate a continuous phenotypic gradient under 18 °C and short photoperiod conditions, demonstrating considerable plasticity [17]. Humidity also plays a critical role in egg hatching success [18]. Light intensity further modulates diapause induction and larval diurnal behavior (such as feeding) through photoperiodic regulation, thereby influencing energy accumulation and developmental efficiency [19]. The phenology of host plants is driven by temperature and photoperiod, thereby closely linking butterfly development to climatic factors [20]. For example, artificial nighttime lighting doubles the feeding frequency of *D. plexippus* larvae without altering pupal weight or development time [21]. In *Pararge aegeria* (Linnaeus, 1758), moderate light levels delay larval development, but photoperiod during the pupal stage serves as a primary cue for diapause induction [22]. Temperature and light also interact to shape developmental outcomes: temperature accelerates development but reduces body size, whereas photoperiod primarily affects body size by altering growth duration [23]. Despite these advances, most studies remain restricted to single-factor or two-factor analyses, underscoring the need to examine dynamic interactions among light, temperature, and humidity to fully elucidate insect developmental plasticity.

*Telchinia issoria* (Hübner, 1819) [24], historically treated under the genus *Acraea* (as *Acraea issoria*) until recent taxonomic revision, is a butterfly species that utilizes plants of the Urticaceae family as hosts. *T. issoria* is the only species in the *Telchinia* genus found in the Oriental region. It has the typical physical features of its genus [25]. Recent records from Uttar Pradesh and Bihar, India, confirm its presence there. These findings suggest its range in the Indian subcontinent may still be expanding [26]. The study states that for butterflies, spreading to new areas and forming new populations is often directly linked to whether their host plants are available. This is a key factor for their successful establishment [27]. While *T. issoria* is not yet considered a major economic pest, its ecological adaptability and potential to spread are noteworthy. A clear warning comes from a related species, *Acraea terpsicore* (Linnaeus, 1758). Since the 1980s, this butterfly has moved from its native South Asia and established populations in Southeast Asia (e.g., Thailand, Vietnam). It has even reached northern Australia [28]. Following this example, *T. issoria* may also have significant potential to expand its range and establish new populations.

Its life cycle consists of four stages: egg, larva, pupa, and adult. The larvae feed primarily on ramie (*Boehmeria nivea* (L.) Gaudich., 1830), a perennial plant belonging to the Urticaceae family. This species is an important natural fiber crop and is widely distributed across tropical, subtropical, and temperate regions [29]. Adults exhibit slow flight and conspicuous aposematic coloration: the wings have a yellowish-brown ground color with broad black marginal bands, and the hindwings feature triangular arrangements of gray-white patches. These traits make *T. issoria* a widely recognized model species in mimicry studies [30,31]. The larval and adult stages represent the most metabolically active phases of its life history. Larvae require extensive feeding to sustain growth and development, whereas adults engage in energetically demanding activities such as flight, mate searching, and reproduction, rendering them highly sensitive to environmental fluctuations [32]. Although the pupal stage is a sessile metamorphic phase, it remains vulnerable to various environmental conditions [33].Therefore, this study selected *T. issoria* as the focal species. It is a common butterfly, exhibiting typical Lepidopteran traits, and its full life cycle is easily maintained and observed under controlled laboratory conditions. Currently, systematic quantitative data on its life history and environmental responses remain scarce. This research aims to address this fundamental data gap through precise measurements.

Currently, knowledge of this species is largely limited to descriptive records of adult morphology and distribution. There is a lack of systematic, quantitative studies on the relationships between its life history traits and environmental factors such as light, temperature, and humidity. To fill this gap, this study systematically quantifies three key aspects of *T. issoria’s* life history under controlled laboratory conditions: (1) the complete egg-to-adult developmental timeline with continuously logged environmental data; (2) stage-specific survival rates from a cohort of 1418 individuals; and (3) correlations between stage-specific developmental durations and three abiotic variables (temperature, humidity, and light intensity) recorded at 10 min intervals. This represents the first systematic quantitative assessment of life history traits in this species. Through this approach, we establish a foundational dataset for this species, clarify its correlations with these abiotic factors, and highlight the ecological significance of interacting light–temperature–humidity regimes.

## 2. Materials and Methods

### 2.1. Cultivation of Host Plant

The host plant, ramie, was cultivated and used for experiments in a greenhouse at Anhui University, Anhui Province, from April 2024 to January 2025. Plant materials consisted of 181 field-collected individuals and 200 artificially cultivated seedlings. A combination of potted (*n* = 200) and field-cultivated (30 m^2^) plants was employed to meet the leaf supply demands for adult rearing in insect cages and larval feeding, respectively. All plants remained healthy, pest-free, and vigorous throughout the experimental period.

### 2.2. Rearing of Telchinia issoria

Based on its population dynamics and biology, we sampled *T. issoria* at Fuxi Valley, Huangshan City (30.0615° N, 118.1620° E). Sampling occurred on two dates during the peak activity period in September 2024. Specimens were collected across three developmental stages: newly laid eggs (immediately after oviposition), pre-hatching eggs (with translucent chorions showing distinct larval outlines), and second-instar larvae (with exuviae from the first ecdysis present on host leaves). To establish a laboratory cohort, we collected six replicate samples from the field. These samples comprised two groups each of pre-hatching eggs (150 and 100 eggs), newly laid eggs (263 and 273 eggs), and second-instar larvae (402 and 230 individuals), totaling 1418 specimens. All samples were duplicate field collections. Thus, these groups represent different batches of starting material used to establish the laboratory cohort, with no additional experimental treatments applied. During collection, branches of *B. nivea* bearing egg clusters were excised with sterilized pruning shears. To preserve tissue viability, the cut branch ends were immediately wrapped in sterile filter paper moistened with a nutrient solution. This solution was prepared by 2000-fold dilution of a commercial fertilizer (Zhonggu Mid-element Fertilizer) in water, supplying key nutrients (N, P, K, Ca, Mg, B, Zn, Mo). Processed branches were then transported in portable hydroponic chambers to the laboratory, where the nutrient solution was periodically replaced under controlled conditions to monitor eclosion progression [34].

Newly collected and newly hatched larvae were transferred to standardized transparent rearing containers (26 cm × 15 cm × 12 cm) equipped with ventilation ports. Each container was systematically labeled with waterproof tags to indicate the identification number, collection date, and larval instar. A progressive feeding regimen was applied: 1st–3rd-instar larvae received fresh *B. nivea* leaves once daily; 4th–6th instars twice daily; and 7th–8th instars 3–4 times daily. To ensure leaf quality, *B. nivea* branches were cut diagonally at a 45° angle with sterilized scissors and immediately transferred to hydroponic tubes containing nutrient solution. Standardized maintenance included daily removal of frass, dead individuals, and food residues, followed by disinfection of container interiors with sterile gauze saturated in 75% ethanol. Weekly sterilization was performed by temporarily transferring larvae to backup containers. The primary containers were then thoroughly disinfected with 0.1% sodium hypochlorite solution, rinsed, and air-dried. Larval density was strictly regulated according to developmental stage: ≤50 larvae per container for 1st–3rd instars, ≤30 for 4th–6th instars, and ≤15 for 7th–8th instars. Containers were subdivided as needed to ensure adequate activity space per larva. Adults were immediately transferred to pre-prepared rearing cages upon eclosion. These cages contained cultivated *B. nivea* host plants to simulate natural habitats and provide space for activity and oviposition. Adults were supplied daily with a 1:10 (*v*/*v*) honey–water solution presented in feeding dishes placed at accessible cage locations. The solution was replaced regularly to maintain freshness and prevent fermentation.

### 2.3. Life History Traits

During the egg stage, egg coloration, morphology, and developmental status were regularly observed and documented, with oviposition and eclosion dates precisely recorded to calculate incubation duration. In the larval stage, daily monitoring focused on feeding behavior, activity patterns, and morphological changes (e.g., body coloration and length). The timing of each ecdysis was recorded to track changes in larval instars and determine the duration of each instar. When larvae reached the eighth instar and ceased feeding in preparation for pupation, pupation dynamics were closely observed. Throughout the pupal stage, pupal morphology, pupation date, and eclosion date were recorded to calculate pupal duration. After eclosion, adults were transferred to host plant rearing cages, and sex and precise longevity were recorded for each adult to complete the characterization of the entire life cycle.

### 2.4. Morphometric Measurements

Ten larvae were randomly selected from each rearing container for morphological measurements. Larval body length was measured across all instars (1st–8th) daily at 9:00 a.m., prior to container cleaning and host plant replacement. Measurements were taken when the larvae were in a natural resting state with their bodies fully extended. A digital vernier caliper (Deli, Ningbo, China; Model DL91150; accuracy: 0.01 mm) was used to measure from the anterior end of the head to the posterior end of the abdomen along the body axis. All values were recorded, and care was taken to avoid physical contact with the larvae to ensure accuracy and consistency.

Due to their small size and fragility, early-instar larvae (1st–3rd) were excluded from measurements to avoid injury or mortality from mechanical disturbance; therefore, head capsule width and body weight were not recorded for these instars. Only 4th–8th-instar larvae were used for head capsule width and body weight measurements. Head capsule width was measured at the widest point of the head, typically between the compound eyes, using a digital vernier caliper held perpendicular to the body axis to prevent compression or deformation. Body weight was determined using a precision electronic balance (OHAUS, Changzhou, China; Model NVE222ZH; accuracy: 0.01 g) with weighing paper to minimize friction-induced injury. The weighing paper was placed on the balance tray and tared to zero before larvae were gently transferred with a soft brush to avoid direct handling. Measurements were recorded once the larva remained still and the balance reading stabilized. Larvae were immediately returned to their rearing containers after measurement.

For adult butterflies, forewing length was used as a proxy for adult body size [35]. Using a digital vernier caliper, the distance from the base of the wing at the starting point of the Sc vein to the wing apex (the longest straight-line distance) was measured. All adults were measured, with three independent measurements taken for each individual. The arithmetic mean of these replicates was calculated as the final forewing length.

### 2.5. Environmental Parameter Measurement

This study employed an observational design to investigate the relationship between the developmental duration of *T. issoria* and environmental variables under simulated natural fluctuations. The individual larva served as the independent observational and statistical unit. A total of 1418 individuals were tracked continuously, systematically recording the complete developmental process and the duration of each stage from hatching until death or adult eclosion, thereby constructing an individual-level dataset for analyzing developmental timing and environmental responses. During laboratory rearing, key environmental parameters (temperature, humidity, and light intensity) were maintained using a non-constant control mode, allowing them to fluctuate within predefined ranges to simulate natural ecological conditions. Environmental conditions were strictly controlled as follows: (1) temperature was maintained within a set range of 10–30 °C using air conditioning; (2) relative humidity was regulated within a set range of 30–80% RH using humidifiers; (3) a fixed 14 h light: 10 h dark photoperiod was applied, with natural light as the primary source. Light intensity varied dynamically from 0 to 1.1 Klux (0–1100 Lux). To ensure photoperiod stability, a supplemental lighting threshold was set at 50 Lux; LED lights were activated when natural light remained below this level.

Environmental parameters were continuously monitored and logged at 10 min intervals using a multi-parameter environmental monitor (Model TM-THLC-4G, Xuzhou Tengmao Refrigeration Co., Ltd. Xuzhou, China; accuracy: ±0.2 °C for temperature, ±3% RH for humidity, ±5% ± 10 lux for light intensity), enabling 24/7 automated data collection. To ensure that measurements reflected the actual microclimate experienced by the insects, the probes for temperature, humidity, and light were placed directly inside the rearing containers.

### 2.6. Statistical Analysis

Data were organized and preprocessed using Microsoft Excel (Microsoft Corp., Redmond, WA, USA). Normality of developmental duration (*n* = 36–767) and larval morphological indicators (*n* = 166–1157) was tested with the Kolmogorov–Smirnov (K–S) test. Except for fourth-instar body length (D = 0.040, *p* = 0.074) and sixth-instar head-capsule width (D = 0.038, *p* = 0.062), all variables deviated significantly from a normal distribution (*p* < 0.05). Because most data did not meet the assumptions for parametric tests, stage-specific developmental duration and larval morphological data are presented as median and interquartile range (IQR). The IQR, calculated as the difference between the 75th and 25th percentiles, was used to evaluate data dispersion.

Differences in larval morphological traits (head capsule width, body length, and body weight) across instars were analyzed using the Kruskal–Wallis (K–W) rank-sum test (a non-parametric test chosen because data were not normally distributed), which showed a significant overall effect (*p* < 0.001). Post hoc pairwise comparisons between instars were then conducted with Dunn’s test, and the false discovery rate (FDR) was controlled using the Benjamini–Hochberg (B–H) method to ensure statistical reliability. The overall effect size for the K–W test was estimated using epsilon-squared (*ε*^2^). This approach clarified how larval morphological characteristics varied across instar stages. Sexual dimorphism in adult body size was assessed based on forewing length. After verifying normality (Shapiro–Wilk test) and homogeneity of variances (Levene’s test) for forewing length data, an independent samples *t* test was used to compare the sexes (selected because the data met parametric assumptions), with the significance level set at α = 0.05. Cohen’s d was also calculated to estimate the effect size of the observed differences.

Differences in stage-specific developmental durations were assessed with the K–W rank-sum test (again used due to non-normal data), which was significant (*p* < 0.001). Dunn’s post hoc test was then used for pairwise comparisons between stages, with the FDR controlled via the B–H method to ensure statistical reliability. The overall effect size was similarly reported as epsilon-squared (*ε*^2^). This procedure revealed the pattern of variation in developmental duration across stages. Stage-specific survival rates were calculated as (number of surviving individuals at a given stage/number of individuals at the start of that stage) × 100%. To compare stage-specific survival rates across developmental stages, Pearson’s chi-square test was applied to the complete dataset (*n* = 1418) (appropriate for comparing proportions across categorical groups), with Cramér’s V computed as an effect-size measure. Adjusted standardized residuals (critical value |ASR| > 1.96) were examined to determine the stages that contributed to the overall significant difference. Before analysis, the key assumption of the chi-square test—that all expected cell frequencies were ≥5—was confirmed.

First, environmental data recorded at 10 min intervals are aggregated into daily means. For each individual, these daily means are then averaged across the specific start and end dates of each developmental stage to obtain stage-specific environmental values. For the correlation analysis between developmental duration and environmental factors, we used the individual larva as the unit of analysis. However, because larvae were reared in shared containers, their environmental exposures were not independent. Therefore, this analysis is exploratory and aims to describe patterns within the rearing cohort; its results cannot support causal inference at the container or population level. The normality of stage-specific durations (larval, pupal, and adult) and their associated environmental factors (temperature, humidity, and light intensity) was examined with the Shapiro–Wilk test. All variables were non-normally distributed (*p* < 0.05). Consequently, Spearman’s rank correlation was employed to evaluate the relationships between developmental duration and environmental factors (selected as a non-parametric measure of monotonic association for non-normal data). Following the guidelines for interpreting the absolute value of Spearman’s rank correlation coefficient (|r_s_|) [36], coefficients were interpreted as: 0.00–0.19 (very weak), 0.20–0.39 (weak), 0.40–0.59 (moderate), 0.60–0.79 (strong), and 0.80–1.00 (very strong). All statistical analyses were conducted using IBM SPSS Statistics for Windows v27 (IBM Corp., Armonk, NY, USA), and data visualizations were created using Origin 2024 (Origin Lab Corp., Northampton, MA, USA).

## 3. Results

### 3.1. Morphological Features of Telchinia issoria

**Figure 1:**
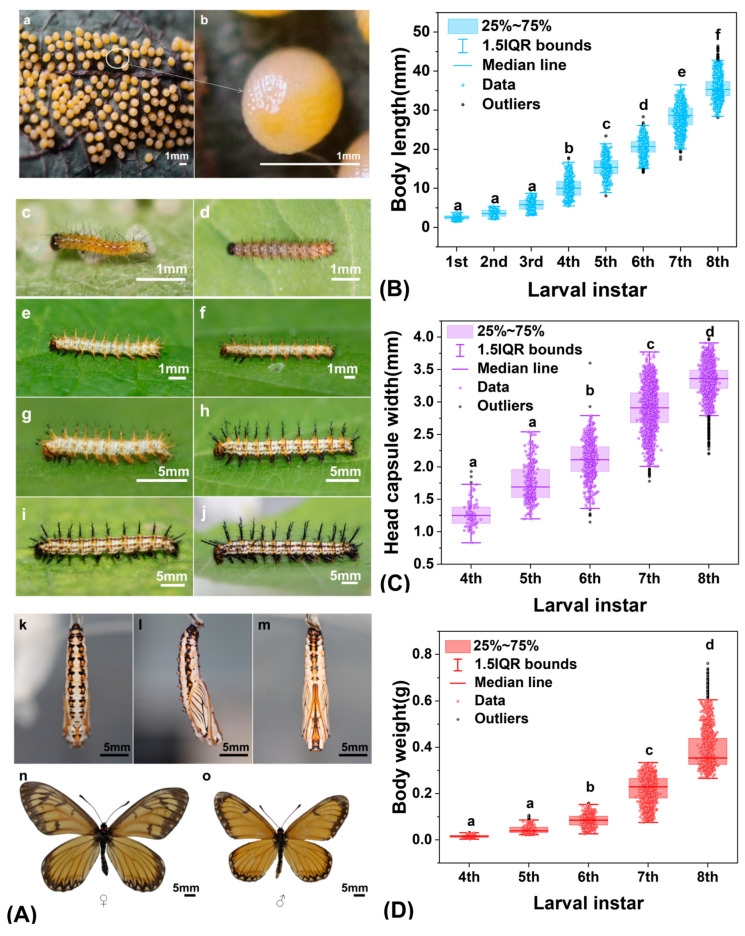
Life history and larval morphological development of *Telchinia issoria*. (**A**) Life stage illustrations. **Eggs**: (**a**) egg cluster, (**b**) individual egg; **Larvae**: (**c**) 1st instar, (**d**) 2nd instar, (**e**) 3rd instar, (**f**) 4th instar, (**g**) 5th instar, (**h**) 6th instar, (**i**) 7th instar, (**j**) 8th instar; **Pupa**: (**k**) ventral view, (**l**) lateral view, (**m**) dorsal view; **Adults:** (**n**) female, (**o**) male. (**B**–**D**) Morphometric distributions across larval instars (sample sizes per instar ranged from *n* = 166–767). (**B**) Body length distribution across 1st–8th instar larvae (*H*(7) = 5527.294, *p* < 0.001). (**C**) Head capsule width distribution in 4th–8th-instar larvae (*H*(4) = 3200.418, *p* < 0.001). (**D**) Body mass distribution in 4th–8th-instar larvae (*H*(4) = 4213.811, *p* < 0.001). K–W test with Dunn’s post hoc comparison, B–H adjusted *p* < 0.05. Significant differences among stages are denoted by distinct lowercase letters above boxplots.

The life cycle of *T. issoria* comprises four stages: egg, larva, pupa, and adult (Figure 1A(a,b)). The eggs are ovate to spherical and are typically deposited in clusters on the abaxial surface of host plant leaves. The larvae pass through eight instars (Figure 1A(c–j)). Instars 1–3 exhibit minimal morphological differentiation and display gregarious silk-nesting behavior. From the fourth instar onward, body segmentation becomes distinct (head, three thoracic segments, and ten abdominal segments), and the larvae shift to solitary behavior with enhanced mobility. The pupae are suspendarian (Figure 1A(k–m)). They have a smooth, dorsally curved, and conical shape. The cuticle is yellowish-white with black markings, and the wing venation is prominent. Adults (Figure 1A(n,o)) possess clavate antennae and an orange-red circular patch on the neck, with a slender abdomen. Wings are yellowish-brown: forewings feature broad black marginal bands and white spots, whereas hindwings show serrated black marginal lines and a medial yellow band on the ventral side.

Sexual dimorphism is evident: independent samples *t*-test revealed that females have significantly longer forewings than males (*t*(34) = 4.206, *p* < 0.001). The mean values were 34.04 mm (SD = 3.22, *n* = 15) and 29.90 mm (SD = 2.67, *n* = 21), respectively, resulting in a mean difference of 4.14 mm. The effect size was extremely large (Cohen’s d = 1.43). This distinct sexual dimorphism likely reflects adaptations related to their reproductive ecology. Longer forewings in females may enhance flight efficiency and endurance, supporting their search for suitable host plants and dispersed oviposition sites, thereby increasing reproductive success. In contrast, the relatively shorter wings in males may provide greater maneuverability, advantageous for courtship and territorial defense. In terms of wing patterns, females bear three transverse black bands and one submarginal stripe on the forewings, whereas males have only a single discal black band.

Larval body length increased steadily from the 1st to the 3rd instar and then rose sharply after the 4th instar, accounting for most of the variation in this trait (*ε*^2^ = 0.875; Figure 1B). Similarly, instar explained substantial variation in head capsule width (*ε*^2^ = 0.615) and body weight (*ε*^2^ = 0.810; Figure 1C,D). Both traits increased markedly after the 4th instar, with no significant difference between the 4th and 5th instars.

Together, these three morphometric traits outline a developmental trajectory of “slow early growth followed by accelerated growth after the 4th instar,” with very large effect sizes (all *ε*^2^ > 0.6). This pattern may reflect a shift in resource allocation: the early slow phase possibly supports tissue differentiation and organogenesis, whereas the later rapid phase likely facilitates the accumulation of biomass and energy reserves required for metamorphosis.

### 3.2. Telchinia issoria Life History Trait

**Figure 2:**
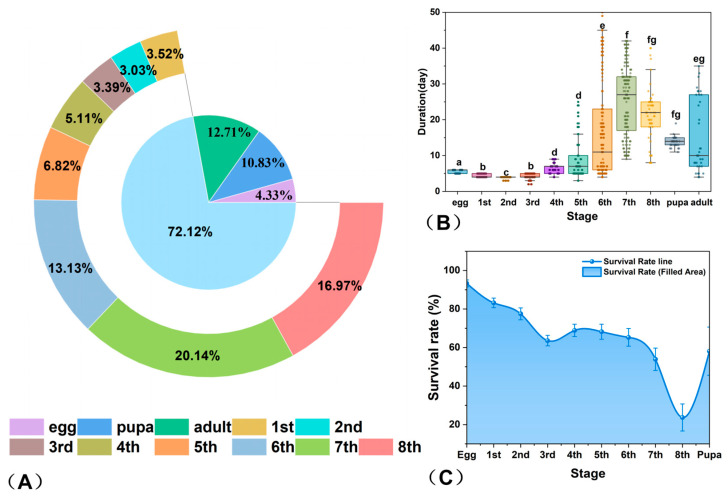
Stage-specific characteristics of developmental duration and survival rate dynamics in *T. issoria*. (**A**) Proportional temporal allocation across developmental stages. (Percentages are rounded to two decimal places and may not total 100%.) (**B**) Stage-specific variation in developmental duration. Significant differences among stages are denoted by distinct lowercase letters above boxplots (K–W test with Dunn’s post hoc comparison, B–H adjusted *p* < 0.05). (**C**) Survival rate variation per developmental stage. The total sample size was *n* = 1418.

Developmental time allocation (Figure 2A) was dominated by the larval stage, within which late instars (6th–8th) accounted for a significantly greater proportion than early and middle instars (*p* < 0.05). Stage durations (Figure 2B, Table S1) increased progressively with instar, with late instars showing marked prolongation relative to earlier stages (*p* < 0.05). Pupal duration slightly exceeded that of the adult stage, whereas the egg stage was the shortest. Summation of the median duration across all stages gave an estimated total generation time of approximately 117.0 days, which serves as a central-trend estimate of development time.

The significant effect of developmental stage on duration (K–W test, *p* < 0.001) reflects a stage-specific resource allocation strategy in larval life history. Early instars (1st–3rd) were brief and stable, consistent with investment in foundational development, whereas late instars (6th–8th) were markedly prolonged, corresponding to biomass accumulation for metamorphosis and reproduction. The lack of significant differences between certain consecutive stages (e.g., 4th–5th, 7th–8th, pupa–adult) suggests functional plateaus, indicating a rhythmic balance in energy allocation.

Survival analysis revealed a significant stage-dependent decline across development (χ^2^ = 426.63, *df* = 9, *p* < 0.001; Figure 2C, Table S2). Egg survival was highest (95.15%), while the late larval instars, particularly the 8th instar (30.72%), exhibited the sharpest mortality increase. Adjusted residual analysis confirmed that survival shifted from significantly above expectation in early stages to significantly below expectation from the 3rd instar onward, with the 4th instar and pupal stage as transient exceptions that did not deviate from expected values.

Overall, the larval period exhibits the most concentrated survival risks and pronounced fluctuations. The eighth instar emerges as the critical bottleneck, a stage characterized by both maximum energy reserves and peak physiological risk, ultimately determining the success of the larval-to-pupal transition.

### 3.3. The Effects of Environmental Factors on the Life History of Telchinia issoria

**Figure 3:**
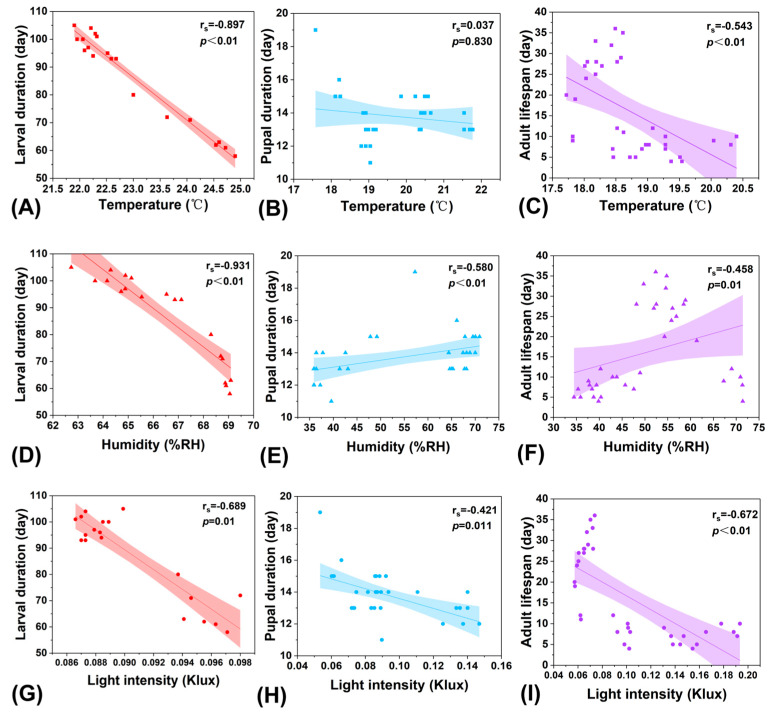
Matrix of Spearman rank correlation analyses between environmental factors (temperature, humidity, light intensity) and developmental duration across distinct life stages of *T. issoria*. (**A**) Temperature−larval duration (*r_s_* = −0.879, *p* < 0.01, *n* = 19); (**B**) Temperature−pupal duration (*r_s_* = 0.037, *p* = 0.83, *n* = 36); (**C**) Temperature−adult duration (*r_s_* = −0.543, *p* < 0.01, *n* = 36); (**D**) Humidity−larval duration (*r_s_* = −0.931, *p* < 0.01, *n* = 19); (**E**) Humidity−pupal duration (*r_s_* = 0.580, *p* < 0.01, *n* = 36); (**F**) Humidity−adult duration (*r_s_* = 0.458, *p* = 0.005, *n* = 36); (**G**) Light intensity−larval duration (*r_s_* = −0.689, *p* = 0.01, *n* = 19); (**H**) Light intensity−pupal duration (*r_s_* = −0.421, *p* = 0.01, *n* = 36); (**I**) Light intensity−adult duration *(r_s_* = −0.672, *p* < 0.01, *n* = 36). Solid lines represent linear regression fits with shaded 95% confidence bands, which are included solely to visually illustrate the overall trend of variable changes and do not imply an underlying linear relationship. Statistical inference is based on Spearman’s rank correlation coefficients (*r_s_*) and significance levels (*p*), which do not assume linearity between variables.

Of the 1418 individuals reared in this experiment, 36 successfully developed into adults. All 36 specimens were included in the analyses of adult lifespan and pupal duration, while 17 were excluded from the larval duration analysis due to incomplete larval-stage data, resulting in a final valid sample size of 19 for larval duration. Spearman rank correlation analysis revealed significant associations between environmental parameters and developmental durations. The analysis conducted at the individual level revealed the following trends:

Specifically, larval duration was very strongly negatively correlated with both temperature and humidity, and strongly negatively correlated with light intensity (Figure 3A,D,G). Pupal duration was not correlated with temperature; moderate positive correlations were observed with humidity, and moderate negative correlations with light intensity (Figure 3B,E,H). Adult longevity was moderately negatively correlated with temperature and light intensity, and moderately positively correlated with humidity (Figure 3C,F,I).

This exploratory analysis conducted at the individual level revealed potential associations between temperature, humidity, and light intensity within the shared environment and both developmental timing and adult longevity. Overall, across the larval, pupal, and adult stages, humidity showed the strongest correlations with the larval and pupal periods, while light intensity exhibited the strongest correlation with adult lifespan.

## 4. Discussion

The early larval instars (1st–3rd) of *T. issoria* exhibited slow growth, with minimal variation in body length and other morphological characteristics. A distinct morphological shift occurred at the transition to the 4th and 5th instars, marked by significant increases in body length, mass, and head capsule width compared to earlier stages. Subsequently, developmental pace accelerated sharply from the 6th to the 8th instar, a phase characterized by pronounced enhancement across all measured morphological traits. This staged growth pattern is common among Lepidoptera [37]. The slow growth during early to middle instars may reflect ecological trade-offs associated with early-stage survival and risk reduction. In later instars, rapid growth and nutrient accumulation are positively associated with pupation success, adult body size, and reproductive potential. It should be noted that the interpretation of morphometric data in this study has certain limitations. Measurements of larval head width and weight began from the fourth instar and did not include the first to third instars. As a result, the early stages of growth patterns were not fully captured. The larval stage of *T. issoria* was the longest developmental phase, accounting for 72.05% of the total duration—a pattern consistent with reports for *Acraea* larvae [38].

In holometabolous insects, the larval stage is typically the primary period for growth and development, during which nearly all somatic development occurs. *T. issoria* larvae undergo seven molts, resulting in eight instars. This number is notably higher than the Lepidopteran norm. In contrast, larvae of other species in the families Papilionidae, Lycaenidae, Pieridae, and Nymphalidae—such as *Papilio nireus* (Linnaeus, 1758) [39], *Cheritrella truncipennis* (de Niceville, 1887) [40], *Delias hyparete* (Linnaeus, 1758) [41], and *Dira clytus* (Linnaeus, 1764) [42]—typically undergo only four molts and five instars. The unusually high number of molts and instars in *T. issoria* may be linked to its extended larval duration. Although molting frequency in Lepidopteran larvae is generally fixed, variation has been observed in association with environmental conditions [43]. For example, instar number in *Coenonympha pamphilus* (Linnaeus, 1758) may vary with environmental cues (e.g., short-day-induced diapause), and such variation has been associated with resource use in mild winter climates [44]. Similarly, instar number in *Melitaea cinxia* (Linnaeus, 1758) can vary with temperature and initial larval state; smaller larvae after diapause often add an extra instar, a phenomenon interpreted as phenotypic plasticity correlated with individual fitness under variable conditions [45].

Research data indicate relatively low overall survival rates during the larval stage of *T. issoria*, particularly in the early instars, consistent with previous findings. Field studies on *C. truncipennis* also demonstrated a significant temporal decline in larval numbers, with most mortality occurring in the early instars [40]. Similarly, research on *Zerynthia polyxena* (Denis & Schiffermüller, 1775) and *Euphydryas editha* (Boisduval, 1852) showed that mortality is concentrated in the early larval stages [46,47]. In this study, early-instar (1st–3rd) *T. issoria* larvae exhibited strong gregariousness, frequently spinning silk webs and living communally. In nature, gregariousness and silk-spinning are adaptive, but in the lab, this combination becomes detrimental, causing fatal larval entanglements. As larval development progressed, body size increased, silk-spinning intensified, and mobility was enhanced, resulting in increasingly complex web entanglements. These factors likely explain the rapid decline in survival rates observed in the first three instars. Aggregative and silk-spinning behaviors are well-documented in Lepidoptera. *Chlosyne lacinia* (Geyer, 1837) larvae enhance survival and development through collective defense and cooperative feeding [48], while *M. cinxia* maintains aggregation across four life stages with group size positively influencing survival [49]. Kin-based aggregation occurs, where siblings cluster more readily, maintained by transient leaders [50]. Silk-spinning serves nest construction in *Thaumetopoea pityocampa* (Denis & Schiffermüller, 1776), varying by sex, size, and instar but remaining crucial for survival [51], and aids climbing in at least 13 butterfly species for predator evasion and host plant access [52]. Field observations confirm *T. issoria* also uses silk for climbing back to host plants.

Collectively, these findings suggest that the aggregative and silk-spinning behaviors of early-instar *T. issoria* larvae are adaptive traits that enhance survival under natural conditions. However, under laboratory rearing conditions, these behaviors fail to confer adaptive advantages and instead contribute significantly to the low survival rates observed in the first three instars. Beginning at the 4th instar, larvae adopt a solitary lifestyle, and survival rates gradually improve. However, survival begins to decline again after the 6th instar and drops sharply during the 8th instar. The concomitant rapid increases in body length, head capsule width, and body mass in these later instars may underlie elevated molting failure rates, particularly the exceptionally high failure rate during larval–pupal ecdysis in the 8th instar.

Prior research has shown that larger larval body size is associated with increased complexity and risk during molting. First, a lower surface-area-to-volume ratio in larger larvae has been linked to greater physical stress on the old cuticle and higher physiological demands, including respiration and water balance; these factors together are considered to contribute to the overall challenge of molting [43]. Second, larger body size is accompanied by more extensive chitin synthesis, breakdown, and reorganization, which place greater demands on metabolic and regulatory systems. Disruptions in these processes—such as abnormalities in chitin metabolism—have been associated with molting defects or failure [53]. *T. issoria* has the potential to expand its range and establish new populations. Given the notably low survival rate (only 30.72%) of its eighth-instar larvae and their role as a key developmental stage, this instar may represent a particularly vulnerable phase in the species’ life cycle, and thus could be of potential interest for population monitoring or management strategies.

Lepidopteran insects are regarded as ideal models for investigating the ecological impacts of climate change due to their sensitivity to environmental variation [54]. In this study, the developmental trajectory of *T. issoria* exhibited strong associations with environmental conditions, with stage-specific correlation patterns for key abiotic factors. The larval development period showed a strong negative correlation with both mean temperature (*r_s_* = −0.879, *p* < 0.01) and mean humidity (*r_s_* = −0.931, *p* < 0.01). This pattern is consistent with the known physiological characteristics of Lepidoptera, which rely on external heat sources for thermoregulation [55]; higher temperatures are generally linked to accelerated metabolism and development, potentially shortening the larval period [56]. The observed correlation with humidity may reflect its influence on larval water balance, cuticle permeability, or food utilization efficiency, as extreme humidity levels have been associated with metabolic disruption and developmental delays in prior studies [57]. In contrast, the pupal development period showed a moderate positive correlation with mean humidity (*r_s_* = 0.580, *p* < 0.01), suggesting that under higher humidity conditions, pupal development may be slightly prolonged—possibly in relation to hydration needs during metamorphic tissue reorganization. Nevertheless, the specific physiological mechanisms underlying this association remain to be clarified and warrant further investigation.

Adult longevity showed a strong negative correlation with mean light intensity (*r_s_* = −0.672, *p* < 0.01), consistent with prior observations linking light intensity to adult behavior and multimodal signaling phenotypes in Lepidoptera [58,59]. This correlation aligns with previous reports that higher light intensity is associated with shorter adult lifespan, possibly reflecting increased activity and energy expenditure, though causal mechanisms require experimental validation. In *T. issoria*, adult longevity was also moderately negatively correlated with mean temperature (*r_s_* = −0.543, *p* < 0.01), a pattern similarly documented across multiple Lepidopteran species [60]. Simulation studies have indicated that a 3 °C increase in mean temperature is associated with shortened larval and pupal development and reduced adult lifespan [61]. Taken together, these correlations suggest that continued warming could correspond to shorter generation times in *T. issoria*. While such a shift may, in theory, influence population growth potential or habitat suitability, direct assessments of demographic outcomes and range dynamics are needed to evaluate these possibilities. As correlational evidence, the present findings offer physiological indicators that may help inform predictions of species responses and early warning frameworks, but they do not establish causal links.

A key limitation of our experimental design is that larvae were reared in shared containers, resulting in a lack of environmental variation within each container. Consequently, the correlative analyses performed at the individual level are exploratory and must be interpreted with caution. They describe patterns within the cohort but cannot support the same level of inference as an analysis with containers as independent experimental units. Furthermore, the environmental factors of temperature, humidity, and light were not fully separated or independently controlled through a factorial experimental design. This led to natural covariation among these factors. Therefore, the correlations reported reflect the combined effects of multiple environmental variables under natural conditions.

Most existing research has examined associations between single environmental factors and adult stages, often overlooking early life stages. To better understand how environmental conditions relate to butterfly development, future studies could integrate multiple variables under settings that more closely resemble natural habitats and assess their combined associations under dynamic fluctuations [62]. Given the observational nature of our study, the stage-specific sensitivity patterns identified here provide preliminary insights that may guide future work: for instance, investigating interactive relationships among multiple environmental factors during key developmental windows, further exploring the potential ecological role of growth-strategy shifts in response to climate variability, and examining whether vulnerabilities of specific developmental stages could, with additional validation, inform targeted management approaches.

## 5. Conclusions

This study characterizes the life history traits of *T. issoria* by documenting its complete development from egg to adult and identifying stage-dependent variations in survival and environmental sensitivity. The egg stage exhibited the highest survival, in contrast to the markedly reduced survival observed in the eighth-instar larvae. Key abiotic factors may exhibit stage-specific association patterns across different developmental stages: larval duration showed strong negative correlations with both temperature and humidity; pupal duration correlated positively with humidity but negatively with light intensity; and adult longevity was shortened by both elevated temperature and increased light exposure. These results underscore the central role of the larval stage in the species’ life cycle and its phenotypic plasticity under environmental variation, yielding key insights into adaptive strategies of Lepidoptera under environmental stress.

## Data Availability

All data are available in the open figshare repository, and the link to the data is https://doi.org/10.6084/m9.figshare.31123093.

## Supplementary Materials

The following supporting information can be downloaded at: https://www.mdpi.com/article/10.3390/insects17020216/s1, Table S1: Duration of developmental stages in *Telchinia issoria* (Median and IQR); Table S2: Survival rates of *Telchinia issoria* across developmental stages.
