# Supplementary material for: Life History Traits and Developmental Duration of the Yellow Coster Telchinia issoria Hübner, 1819 (Lepidoptera: Nymphalidae) Under Laboratory Conditions
Source: Insects. 2026 Feb 19;17(2):216. doi: 10.3390/insects17020216 (PMC12942303; doi:10.3390/insects17020216)
Supplement: Supplementary file 1 [file insects-17-00216-s001.zip › insects-4139347-supplementary.pdf]

Table S1. Duration of developmental stages in *Telchinia issoria* (Median and IQR)

Stage	Number	Duration (days, Median (IQR))
Egg	510	6(5-6) [a]
1st Instar	651	4(4-5) [b]
2nd Instar	525	4(4-4) [c]
3rd Instar	767	4(4-5) [b]
4th Instar	553	7(5-7) [d]
5th Instar	387	7(5-10) [d]
6th Instar	278	11.5(6-30) [e]
7th Instar	166	27(16-32) [f]
8th Instar	51	22(18-25) fg
Pupa	45	14(13-14.75) fg
Adult	36	10.5(7.25-27) eg
Generation period	—	117

Note: The data in this table represent the individual duration of each developmental stage in *Telchinia issoria*. The median and interquartile range (IQR, i.e., the 25th to 75th percentiles) are reported. Different superscript letters (a, b, c…) indicate statistically significant differences at $p < 0.05$ based on the Kruskal-Wallis test followed by Dunn's post-hoc multiple comparisons (shared letters denote no significant difference). The "Generation period" represents the median total duration from egg to adult across all individuals and was not included in the statistical comparisons among stages.

Table S2. Survival rates of *Telchinia issoria* across developmental stages

Stage	Initial Number	Survival Number	Mortality Number	Survival Rate (%)	Survival Residual	Mortality Residual	Significance Level
Egg	536	510	26	95.15	11.8	-11.8	***
1st Instar	760	651	109	85.66	7.9	-7.9	***
2nd Instar	651	525	126	80.65	4.2	-4.2	***
3rd Instar	1157	767	390	66.29	-6.7	6.7	***
4th Instar	767	553	214	72.10	-1.3	1.3	ns
5th Instar	553	387	166	70.00	-2.2	2.2	*
6th Instar	387	278	109	71.83	-1.0	1.0	ns
7th Instar	278	166	112	59.71	-5.6	5.6	***
8th Instar	166	51	115	30.72	-12.9	12.9	***
Pupa	51	36	15	70.59	-0.6	0.6	ns

Note: This table presents the stage-specific survival rate analysis of *Telchinia issoria*. A Pearson chi-square test was first performed, revealing a significant overall difference in survival rate distribution across stages ($p < 0.001$). This was followed by an analysis of adjusted standardized residuals as a post-hoc test to identify stages where observed counts significantly deviated from expected values. The "Adjusted Residuals" column displays the results; an absolute value > 1.96 indicates a significant deviation at $p < 0.05$. Significance marks are based on these residuals: *** p < 0.001, * $p < 0.05$, ns $p \geq 0.05$. Variations in "Initial Number" stem from the pooling of samples initiated at different developmental stages.
